# Supplementary material for: The Systems Biology Research Tool: evolvable open-source software
Source: BMC Syst Biol. 2008 Jun 29;2:55. doi: 10.1186/1752-0509-2-55 (PMC2446383; doi:10.1186/1752-0509-2-55)
Supplement: Additional file 1 — SBRT Archive. An archive of the current version of the Systems Biology Research Tool. [file 1752-0509-2-55-S1.zip › sbrt-1.4.0/doc/developers_guide/api/sbrt/shell/io/class-use/RomoFile.html]

Uses of Class sbrt.shell.io.RomoFile


|  |  |  |
| --- | --- | --- |
| **Overview** \| **Package** \| **Class** \| **Use** \| **Tree** \| **Deprecated** \| **Index** \| **Help** | |  |
| PREV   NEXT | **FRAMES**    **NO FRAMES**     **All Classes** |  |


---


## **Uses of Class sbrt.shell.io.RomoFile**

| Packages that use RomoFile | |
| --- | --- |
| **sbrt.shell.io** | Provides classes and interfaces for reading and writing files. |
| **sbrt.shell.mng.fba** | Provides classes and interfaces for creating Flux Balance Analysis process managers. |

Uses of RomoFile in sbrt.shell.io

| Methods in sbrt.shell.io that return RomoFile | |
| --- | --- |
| `RomoFile<K,V>` | `RomoFileSource.next()`             Returns the next row-oriented map output file with the next name returned by the file name source. |

Uses of RomoFile in sbrt.shell.mng.fba

| Methods in sbrt.shell.mng.fba that return RomoFile | |
| --- | --- |
| `static RomoFile<java.lang.String,ChemRxn>` | `WwFluxomeReducerManager.getOutputFile(java.lang.String fileName)`             Returns a file to which the chemical reactions of a fluxome can be written. |
| `static RomoFile<java.lang.String,ChemRxn>` | `WwFluxomeReducerManager.getOutputFile(java.lang.String fileName, java.io.PrintWriter out)`             Returns a file to which the chemical reactions of a fluxome can be written. |
| `static RomoFile<java.lang.String,Interval>` | `SimpleFluxIntervalManager.getSingleResultOutputFile(ManagerInput<java.lang.String,java.lang.String> input, java.io.PrintWriter out, Fluxome fluxome)`             Returns an empty output file that is suitable for storing a single set of flux interval results. |
| `static RomoFile<java.lang.String,Interval>` | `SimpleFluxIntervalManager.getSingleResultOutputFile(java.lang.String fileName, java.util.Set<java.lang.String> headers, Fluxome fluxome)`             Returns an empty output file that is suitable for storing a single set of flux interval results. |

---


|  |  |  |
| --- | --- | --- |
| **Overview** \| **Package** \| **Class** \| **Use** \| **Tree** \| **Deprecated** \| **Index** \| **Help** | |  |
| PREV   NEXT | **FRAMES**    **NO FRAMES**     **All Classes** |  |


---
